# DISH of the cervical spine causing epiglottis impingement

**DOI:** 10.4103/0971-3026.50831

**Published:** 2009-05

**Authors:** Tommaso Bartalena, Francesco Buia, Alberto Borgonovi, Maria Francesca Rinaldi, Cecilia Modolon, Francesco Bassi

**Affiliations:** Department of Radiology, S. Orsola University Hospital - via Massarenti 9, 40138, Bologna, Italy

**Keywords:** Diffuse idiopathic skeletal hyperostosis, dysphagia, videofluoroscopy

## Abstract

Diffuse idiopathic skeletal hyperostosis (DISH) is a condition characterized by calcification and ossification of ligaments and entheses; it mainly affects the vertebral column. We report the case of a patient with pharyngeal dysphagia and episodic aspiration secondary to DISH involvement of the cervical spine, which had caused alteration in the epiglottic tilt mechanism during deglutition.

Epiglottic tilt is a protective mechanism of the airways, which acts in the pharyngeal phase of deglutition. Prevertebral ossification in patients with DISH may cause mechanical impingement on the epiglottis with resultant incomplete closure of the laryngeal inlet during swallowing, putting the patient at risk for aspiration.

## Case Report

An 83-year-old man was referred to our hospital with a long-standing history of dysphagia to liquids and solid food and episodic aspirations, along with the complaint of a sensation of having a lump in the back of the throat. His symptoms had worsened over the last 3 months. The patient also reported long-standing stiffness of his neck, with limitation of the range of motion.

**Figure 1 (A–C):**
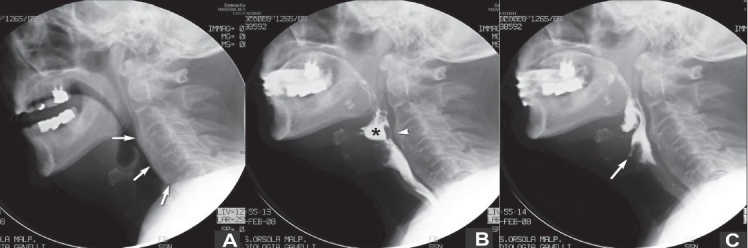
Spot films from a videofluoroscopy swallowing study. Precontrast image (A) shows diffuse thick prevertebral ossification (arrows) of the cervical spine. Intervertebral discs heights are preserved. A congenital C2-C3 block vertebra is seen. The tip of the epiglottis (at rest) is located at the C3-C4 level. Pharyngeal phase of deglutition (B, C). Hyoid bone and laryngeal elevation are seen, but the epiglottis fails to invert because of impingement by the C3-C4 ossification (arrowhead in B). Note pooling of contrast in the valleculae (asterisk in B) and laryngeal penetration (arrow in C)

Precontrast images [Figure 1A] showed diffuse, thick prevertebral ossification extending from C3 downward, which was responsible for a smooth bulge on the posterior wall of the pharynx. A congenital C2-C3 block vertebra was also present. The heights of the intervertebral spaces were preserved. Because of the history of aspiration, videofluoroscopy was performed with the administration of a water-soluble iodinated contrast medium (Gastrografin, 370 mg I/ml; Bayer Schering Pharma, Berlin, Germany). The oral phase of deglutition showed premature leakage of the bolus from the mouth into the valleculae because of age-related deficiency in the tongue volume. In the pharyngeal phase, the epiglottis failed to completely invert because of mechanical impingement due to the prevertebral ossification at the level of C3-C4, and it remained in a semiupright position during bolus passage [Figure 1, B and C]. Premature leakage of oral content into the pharynx and epiglottic tilt failure acted together to bring about laryngeal penetration. The esophageal phase was within normal limits. The radiologic findings were consistent with diffuse idiopathic skeletal hyperostosis (DISH) of the cervical spine causing dysphagia.

Because of his advanced age and cardiac comorbidity, surgery was not considered. The patient was given swallowing training and nonsteroidal anti-inflammatory drugs (NSAIDs), partially relieving his symptoms.

## Discussion

DISH, also known as Forestier disease, is disorder of unknown origin. It was first described by Forestier and Rotes-Querol in 1950.[1] It is characterized by diffuse ossification and calcification of tendons, ligaments, and fasciae in both the axial and the appendicular skeleton. This disease has a predilection for men (65%) and is common in patients over the age of 50, with a prevalence of approximately 15–20% in the elderly population.[2,3] The spinal column is most often affected, with the thoracic spine being most commonly involved (95% of the patients), followed by the lumbar and cervical spines.[4,5]

According to Resnick,[6] the radiographic diagnostic criteria in the spine include: 1) osseous bridging along the anterolateral aspect of at least four vertebral bodies; 2) relative sparing of intervertebral disc heights, with minimal or absent disc degeneration; and 3) absence of apophyseal joint ankylosis and sacroiliac sclerosis.

The clinical manifestations are variable. Some subjects are completely asymptomatic, while others complain of back pain and stiffness and symptoms secondary to encroachment of the bony excrescences on neighboring structures. Pharyngoesophageal and tracheal compression may result in dysphagia, dyspnea, and stridor.[7] The reported incidence of dysphagia in patients with DISH ranges from 0.2–28%; dyspnea is less common.[8] Myelopathy associated with ossification of the posterior longitudinal ligament (OPLL) is another possible complication since OPLL has been described in association with DISH in up to 50% of cases.[9] DISH also predisposes to complications such as iatrogenic mucosal and laryngeal damage during endoscopic procedures and intubation.[10]

Conventional radiography is usually sufficient to confirm the diagnosis of DISH and dynamic videofluoroscopy should be reserved for demonstrating the exact relationship of the cervical spine alterations with the swallow function in those patients who are experiencing dysphagia.[11] CT and MRI may be used to better detect associated findings (e.g., OPLL) and complications (e.g., spinal canal stenosis and compressive myelomalacia).[5]

Dysphagia in patients with DISH involvement of the cervical spine develops mainly because of mechanical compression causing varying degrees of esophageal obstruction, impaired epiglottic motility, and distortion of the laryngeal cartilages. Moreover, chronic compression may result in an inflammatory process in the esophageal wall that can lead to fibrosis and adhesions, with fixation of the esophagus and disruption of the neural plexus.[8] Alterations of the upper cervical spine, especially at the C3-C4 level, may interfere with laryngeal function,[10] while more distal involvement may induce spasm of the upper esophageal sphincter (which is usually located at C5-C6) or esophageal compression.[8,12] In our case, dynamic videofluoroscopy showed mechanical impingement between the prevertebral ossification at C3-C4 and the epiglottis, altering the pharyngeal phase of swallowing and leading to laryngeal penetration. Epiglottic tilt is a laryngeal protective mechanism that acts in the early part of the pharyngeal phase of deglutition to prevent penetration and aspiration of the bolus into the airways during swallowing. Epiglottic tilt occurs as a two-step procedure in the majority of patients. With the first movement, which is passive and induced by hyoid elevation, the epiglottis assumes a horizontal position. With the second movement, which is probably brought about by contraction of the thyroepiglottic muscle, the epiglottis finally reaches the inverted position, closing the laryngeal inlet.[12] Thus, inadequate tilt of the epiglottis may predispose to passage of bolus into the airways and the risk of aspiration pneumonia.

Patients with dysphagia due to DISH may be initially treated conservatively with dietary modifications and swallowing therapy. However, when the dysphagia is severe or when conservative treatment fails, surgical decompression with osteophytectomy via the perioral-transpharyngeal route for the C1 and C2 vertebrae or an anterior cervical approach for the C3 to C7 vertebrae should be considered.[13] Some authors recommend early surgery even when the patient has only mild dysphagia,[14] while others think that surgical intervention should be considered only after trials of medical therapy (NSAIDs, steroids, skeletal relaxants, anti-reflux drugs, etc.) have failed to provide relief.[11]

## Conclusion

Videofluoroscopy is the main tool for investigating deglutition and is indicated in patients with DISH who experience dysphagia since it can provide exact correlation between the anatomical alterations of the cervical spine and functional impairment of the swallowing chain.
